# PReS-FINAL-2102: Comparison of sensitivity and specificity of MAS and HLH diagnostic guidelines in 362 children with MAS complicating systemic JIA

**DOI:** 10.1186/1546-0096-11-S2-P114

**Published:** 2013-12-05

**Authors:** S Davì, F Minoia, AC Horne, E Demirkaya, N Ilowite, A Insalaco, M Ioseliani, M Jelusic-Drazic, MA Kapovic, O Kasapcopur, I Kone-Paut, L Lepore, C Li, S Magni-Manzoni, D Maritsi, D Mccurdy, R Merino, P Miettunen, N Ruperto, A Martini, RQ Cron, A Ravelli, International Investigator Consortium for MAS Diagnostic Criteria

**Affiliations:** 1 Istituto Giannina Gaslini and University of Genova, Genova, Italy; 2 Department of Women's and Children's Health, Childhood Cancer Research Unit, Karolinska Institutet, Karolinska University Hospital Solna, Stockholm, Sweden; 3 Gulhane Military Medical Faculty, Ankara, Turkey; 4 Children's Hospital at Montefiore, Albert Einstein College of Medicine New Hyde Park, NY, United States; 5 Ospedale Pediatrico Bambin Gesu', Rome, Italy; 6 M. Iashvili Children's Central Clinic, Tbilisi, Georgia; 7 University Hospital Centre Zagreb, University School of Medicine, Croatia; 8 Department for pulmology, allergology, clinical immunology and rheumatology, Zagreb, Croatia; 9 Istanbul University, Cerrahpasa Medical Faculty, Istanbul, Turkey; 10 Rhumatologie Pediatrique, Centre de reference national des maladies auto-inflammatoires, Le kremlin Bicetre, Paris, France; 11 Universita' degli Studi di Trieste, IRCCS Burlo Garofolo, Trieste, Italy; 12 Beijing Children's Hospital, Capital, University of Medical Sciences, Beijing, China; 13 2nd Department of Academic Pediatrics, Athens Medical School, University of Athens, Athens, Greece; 14 Department of Pediatrics, Children's Hospital of Orange County, CA, United States; 15 Unidad De Reumatologia Pediatrica, Hospital Universitario La Paz, Madrid, Spain; 16 Department of Pediatrics, Division of Pediatric Rheumatology, University of Calgary, Calgary, Canada; 17 Children's Hospital of Alabama and University of Alabama at Birmingham, AL, United States

## Introduction

Early diagnosis of macrophage activations syndrome (MAS) in systemic juvenile idiopathic arthritis (sjia) may be challenging because it may mimic the clinical features of the underlying disease or be confused with an infectious complication. However, the diagnostic value of the guidelines for hemophagocytic lymphohistiocytosis (HLH) or sjia-associated MAS has seldom been examined.

## Objectives

To investigate the sensitivity and specificity of diagnostic guidelines for HLH and sjia-associated MAS in patients with sjia who developed MAS.

## Methods

The study sample included 362 children with sjia who had MAS (diagnosed and treated as such by the attending physician) and 2 control groups with potentially "confusable" conditions, including active sjia without MAS (n = 404) and a systemic febrile infection requiring hospitalization (n = 345). Diagnostic guidelines for HLH and sjia-associated MAS were applied to all MAS and control patients. Because no patient had NK-cell activity and soluble CD25 determination available and bone marrow aspirate was performed in only a few patients, these 3 criteria were excluded from HLH guidelines. HLH criteria were, therefore, met when at least 4 of the 5 remaining variables were present. Sjia-associated MAS criteria were met when at least 2 laboratory criteria or at least 1 laboratory criterion and 1 clinical criterion were present. Sensitivity and specificity of guidelines in discriminating patients with MAS from control patients were assessed.

## Results

The table shows the comparison of sensitivity and specificity of diagnostic guidelines.

**Table 1:** 

Diagnostic guidelines	MAS vs. Active sjia		MAS vs. Systemic infection	
	
	Sensitivity	Specificity	Sensitivity	Specificity
HLH	0.19	1	0.19	1

Sjia-associated MAS	0.79	0.92	0.79	0.8

## Conclusion

The diagnostic guidelines for sjia-associated MAS revealed strong sensitivity and specificity, whereas HLH guidelines were highly specific, but lacked sensitivity. Sensitivity of HLH was mostly hampered by the excessive stringent threshold for cytopenia and hypofibrinogenemia, and the infrequent occurrence of splenomegaly in patients with MAS.

## Disclosure of interest

None declared.

